# Wood Utilization Is Dependent on Catalase Activities in the Filamentous Fungus *Podospora anserina*

**DOI:** 10.1371/journal.pone.0029820

**Published:** 2012-04-27

**Authors:** Anne Bourdais, Frederique Bidard, Denise Zickler, Veronique Berteaux-Lecellier, Philippe Silar, Eric Espagne

**Affiliations:** 1 Institut de Génétique et Microbiologie, Univ Paris-Sud, UMR 8621, Orsay, France; 2 CNRS, Orsay, France; 3 Institut Génétique et Développement de Rennes, CNRS, UMR 6061, Rennes, France; 4 UEB Université Rennes 1, IFR 140, Faculté de Médecine, Rennes, France; 5 Laboratoire d’Excellence « CORAIL », USR 3278 CNRS-EPHE, CRIOBE, BP 1013, Moorea, French Polynesia; 6 Univ Paris Diderot, Sorbonne Paris Cité, UFR des Sciences du Vivant, Paris, France; University of Wisconsin - Madison, United States of America

## Abstract

Catalases are enzymes that play critical roles in protecting cells against the toxic effects of hydrogen peroxide. They are implicated in various physiological and pathological conditions but some of their functions remain unclear. In order to decipher the role(s) of catalases during the life cycle of *Podospora anserina*, we analyzed the role of the four monofunctional catalases and one bifunctional catalase-peroxidase genes present in its genome. The five genes were deleted and the phenotypes of each single and all multiple mutants were investigated. Intriguingly, although the genes are differently expressed during the life cycle, catalase activity is dispensable during both vegetative growth and sexual reproduction in laboratory conditions. Catalases are also not essential for cellulose or fatty acid assimilation. In contrast, they are strictly required for efficient utilization of more complex biomass like wood shavings by allowing growth in the presence of lignin. The secreted CATB and cytosolic CAT2 are the major catalases implicated in peroxide resistance, while CAT2 is the major player during complex biomass assimilation. Our results suggest that *P. anserina* produces external H_2_O_2_ to assimilate complex biomass and that catalases are necessary to protect the cells during this process. In addition, the phenotypes of strains lacking only one catalase gene suggest that a decrease of catalase activity improves the capacity of the fungus to degrade complex biomass.

## Introduction

Reactive oxygen species (ROS), including superoxide anions (O_2_^.−^), hydroperoxyl radicals (HO_2_^.^), hydroxyl radicals (^.^OH), and hydrogen peroxide (H_2_O_2_) play crucial roles in various aspects of cell physiology. They are constantly generated as by-products of aerobic metabolism and through enzymatic activity [1], [2]. The two main metabolic sources of ROS are the mitochondrial respiratory chain and the peroxisomal fatty acid ß-oxidation pathways [3], [4]. ROS can also be produced by dedicated enzymes, such as oxidases and peroxidases [1]. ROS have been implicated in two major aspects of fungal biology. First, they are involved through the action of NADPH oxidases in signaling developmental processes [5]–[9]. Second in Basidiomycetes where plant biomass degradation is well documented, several ROS producing enzymes, including GMC oxidoreductases, galactose oxidases, copper radical oxidases, quinone reductases, pyranose oxidases and cellobiose dehydrogenases have been implicated in lignin lysis [10]–[13]. However, the enzymology of plant biomass degradation proceeds differently in different fungi, in relation to the enzymes encoded by their genomes. Fungi such as *Pycnoporus cinnabarinus* use laccases, while others like the white rot *Phanerochaete chrysosporium* use several classical ROS-generating oxidative enzymes [14], [15]. Nevertheless, in every case H_2_O_2_ may be produced to degrade cellulose [16], [17].

Although ROS play important roles in fungal biology, their accumulation causes oxidative damage to macromolecules and are thus deleterious for cellular integrity [18]–[20]. The cell primary defense mechanism against ROS is provided by the hydroperoxidases that include the monofunctional catalases and the bifunctional peroxidase/catalase enzymes. They constitute the major defense system against hydrogen peroxide, one of the most frequently occurring ROS [21]–[23]. Both types of catalases are metalloenzymes decomposing H_2_O_2_ to water and molecular oxygen [24], [25].

In order to better understand the role of catalases and consequently the possible functions of hydrogen peroxide during the life cycle of the filamentous fungus *Podospora anserina,* we identified and investigated the role of each of the five catalases present in this fungus. Our data show that catalase activity is specifically required to efficiently assimilate lignocellulose and that the catalase that provides the best protection against peroxide (CAT2), is also the one with the major role during lignocellulose breakdown.

## Results

### Four Catalases and One Peroxidase/Catalase Genes are Present in *P. anserina* Genome

*P. anserina* catalase genes were identified from the genomic DNA sequence (http://podospora.igmors.u-psud.fr; [26] using the *N. crassa* catalase protein sequences (NCU05169; NCU08791 and NCU00355) and the peroxidase/catalase 2 (NCU05770) as reference. While only three catalases and one peroxidase/catalase gene are present in the genome of *N. crassa*, *P. anserina* harbors four catalase and one peroxidase/catalase genes. They were named *CatA* (Pa_7_4240), *CatB* (Pa_7_1610), *CatP1* (Pa_5_8140) and *CatP2* (Pa_7_1060) and the peroxidase/catalase gene with two highly conserved peroxidase domains was named *Cat2* (Pa_6_11240).

Phylogenetic analyses of the fungal catalases indicate that number of enzymes varies among species. While only two catalases are present in the unicellular ascomycete *Saccharomyces cerevisiae* (peroxisomal catalase A and cytoplasmic catalase T), four and five have been characterized in *Neurospora crassa* (*cat-1* to *cat-4*) and *Aspergillus nidulans* (*catA* to *catD* and ANID_08553), respectively. Peroxidase/catalases (Figure S1A) are split into two groups, as previously shown by Zamocky *et al.* [27]. *P. anserina* has only one gene encoding a peroxidase/catalase, belonging to the first group of non-secreted enzymes (Figure S1A). Analysis of monofunctional catalases (Figure S1B) reveals five clades [28]. *P. anserina* CATA exhibits a catalase plus a DJ-1/PfpI domain and is a member of clade A and CATB is in clade B, which includes many putative secreted catalases. Recently, Zintel *et al.* have shown that CATB is secreted [29]. *P. anserina* has no C-type catalase and one catalase in each of the last groups of small-subunit monofunctional enzymes: CATP1 (clade P1) and CATP2 (clade P2).

### *CatA*, *CatP1* and *CatP2* are Down Regulated During Sexual Development

To investigate the role of the five *P. anserina* catalases, expression of their genes was monitored by quantitative real time RT-PCR during both vegetative and sexual phases. Although all five genes are expressed during vegetative growth, differences were observed: *Cat2* and *CatB* genes produced abundant transcripts, while the genes encoding the small-subunit monofunctional catalases (*CatP1* and *CatP2*) were poorly expressed (99 and 116 times less than *Cat2* respectively; p-value<0.001).

**Figure 1:**
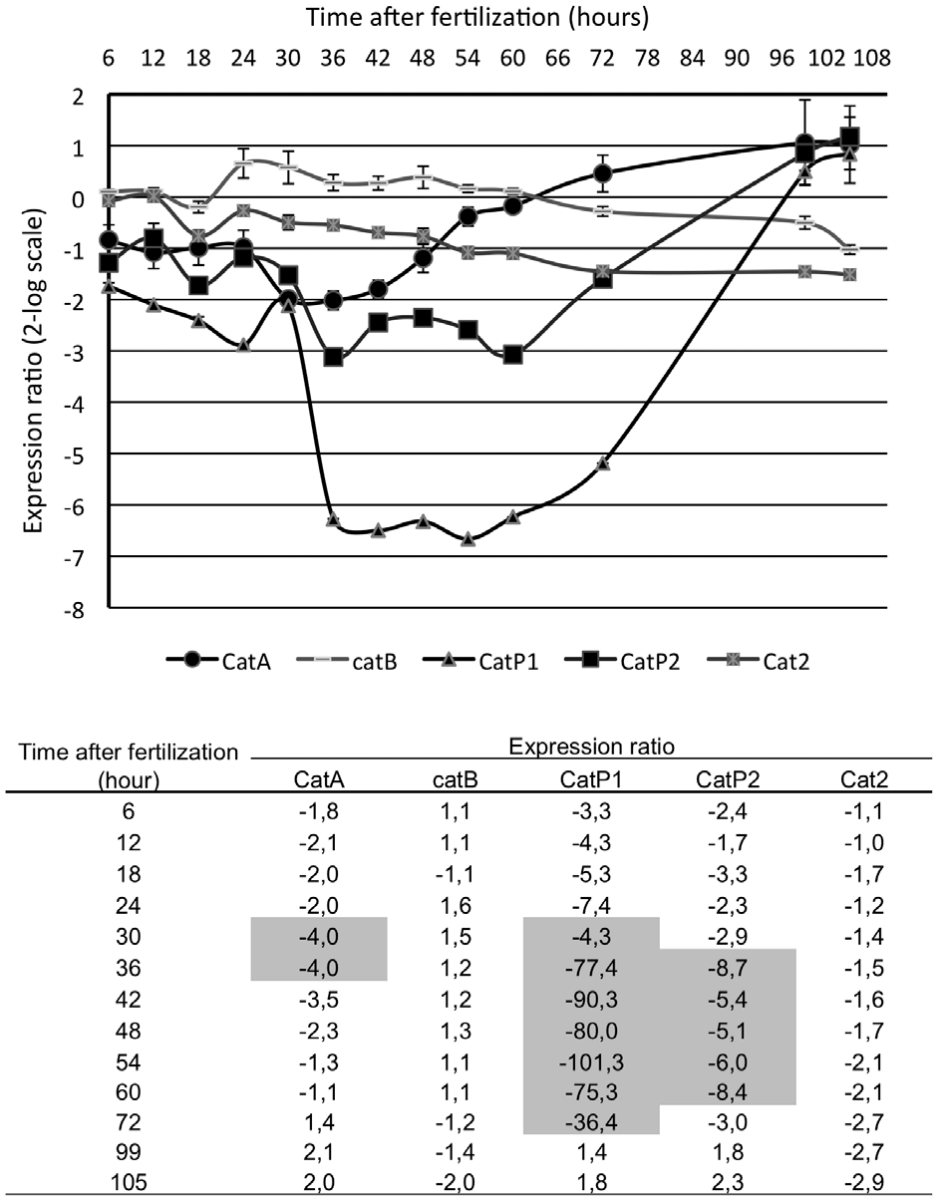
Expression of the five catalases genes at different stages of the sexual cycle. Gene expression was monitored by real time PCR using fertilization as time 0. Changes in gene expression are presented as x-fold change relative to expression at time 0 in 2-log scale. Standard deviations were obtained through three independent experiments and data were normalized by three reference genes (Materials and Methods). The Table below the graph gives the expression ratio of each gene at different times from 6 to 105 hours after fertilization. The significant ratios (p-value = 0.001) are indicated by grey overlapping rectangles.

We next analyzed the expression of these five genes during sexual reproduction, at defined times after fertilization (Figure 1). *P. anserina* fruiting bodies (perithecia) appear 24 hours after fertilization, asci develop from 30 hours on, meiosis occurs from 30 to 70 hours, ascospores form at 72 hours and they are expelled from 96 hours on (Bidard *et al.*, Fungal Genetics Conference, Edinburgh, 2008). Transcription levels at each time point were compared to levels at time 0 of fertilization. Data show that *CatA*, *CatP1* and *CatP2* were significantly down regulated between 30 hours and 72 hours after fertilization, i.e., during ascus (meiocytes) development. The two most down-regulated genes, *CatP1* and *CatP2*, encode the two small-subunit monofunctional catalases and transcription repression was observed during the entire ascus development (Figure 1). Thus, three of the five catalase genes are down regulated during sexual development starting precisely at 30 hours post fertilization, which corresponds to the emergence of the first asci in the fruiting bodies and thus to the time of karyogamy and the first meiotic divisions.

### Although Regulated During the Life Cycle, Catalases are Dispensable During the Vegetative and Sexual Phases

Null alleles were constructed *in vitro* by replacing the five entire catalase protein coding sequence with different resistance markers, hygromycin B (*CatP2* and *CatP1*), phleomycin (*CatA*) and nourseothricin (*Cat2* and *CatB*) (see Materials and Methods). In each case, correct gene inactivation was confirmed by PCR and Southern blot analyses. Because of potential functional redundancy of the catalase genes, we constructed by genetic crosses, all possible combinations of double, triple and quadruple mutants as well as the quintuple mutant completely devoid of its five catalases. The genotypes of all multiple mutants were confirmed by PCR (data not shown).

First, catalase single and multiple mutants were analyzed in standard condition, i.e., on M2 minimal medium at 27°C. For the sake of clarity only the data pertaining to the relevant mutants will be reported here. However, all the other investigated mutants also behaved as wild type. Under this laboratory condition, the single and the quintuple mutants displayed no obvious vegetative defect (see Text S1, Figure S2 and Table S1). They were also able to complete their sexual cycle with the same efficiency as wild type: conidial production, fertilization capacity, perithecium development and ascospore production were identical to those of wild type (see Text S1 for detail and Table S2).

Second, change in temperature condition (11°C or 37°C instead of 26°C) had no effect on the growth of the quintuple mutants on standard medium at 11°C and 37°C (Table S3). Finally, the ability of the mutants to present hyphal interference was evaluated (see Text S1 and Figure S3B). It was shown previously that this defense mechanism against a fungal contestant involves accumulation of ROS and/or redox activity [30]. Note that ROS accumulation and/or redox activity is also observed on unchallenged mycelia, although at lower levels [7]. The quintuple mutant showed no modification of constitutive ROS/Redox activity production nor of hyphal interference, indicating that catalases were also dispensable during interaction of *P. anserina* with other fungi, at least in the laboratory conditions.

### Catalase Activity is Dispensable to Peroxisomal ß-oxidation

**Figure 2:**
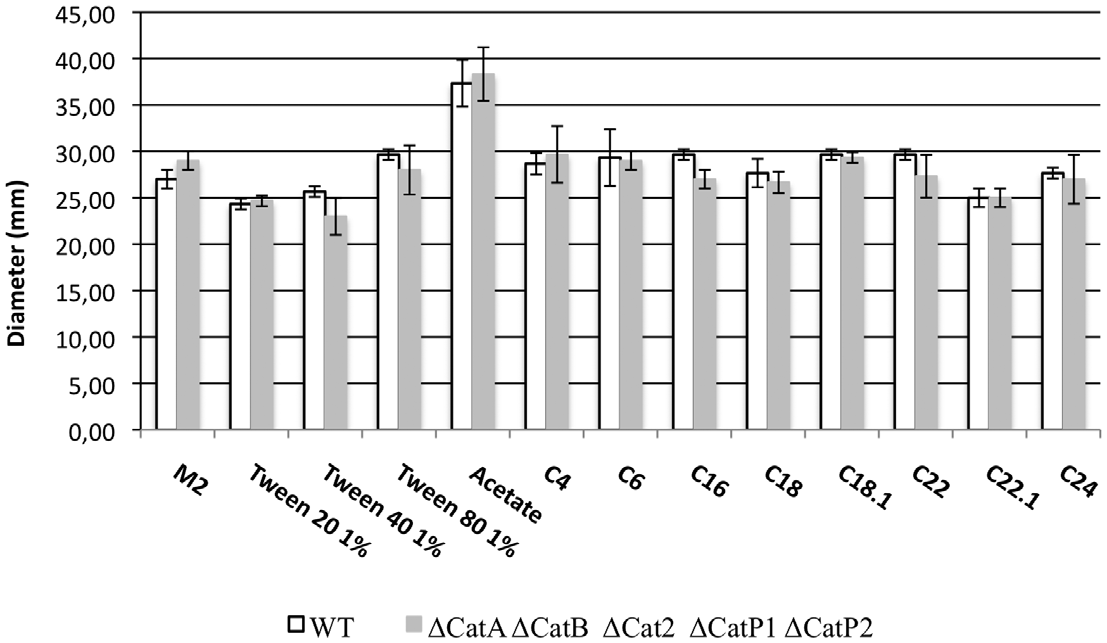
Growing rates of wild type and the quintuple catalase mutants on fatty acids. Thalli diameters (mm) for each strain were measured 72 hours after incubation on solid medium at 27°C. The minimal synthetic medium was used and contains either 0.5% (V/V) dextrin (M2) or various fatty acids as carbon sources: 1% (V/V) Tween20, 1% (V/V) Tween40, 1% (V/V) Tween80, 50 mM acetate, 30 mM butyric acid (C4), 20 mM hexanoic acid (C6), 7.5 mM palmitic acid (C16), 6 mM stearic acid (C18), 6 mM oleic acid (C18∶1), 5 mM behenic acid (C22), 5 mM erucic acid (C22∶1) and 5 mM lignoceric acid (C24). C16, C18, C18∶1, C22, C22∶1 and C24 were solubilized with 0.5% (V/V) tergitol NP40. When required, pH was adjusted to 7. The value for each strain is an average of 6 cultures and error bars represent standard deviation.

We next tested the growth of the mutants on medium containing fatty acids as sole carbon source. The first step of the peroxisomal β-oxidation pathway is usually performed by acyl-CoA oxidase, an enzyme delivering electrons to molecular oxygen and generating the toxic hydrogen peroxide [31]. The growth capacity of the catalase mutants was analyzed on media containing fatty acids with different length and saturation (Figure 2). On the 12 tested carbon sources, all catalase mutants, including the quintuple mutant, exhibited wild-type morphology and growth. This result was expected for acetate or the short chain fatty acid, because their degradation does not require peroxisomal ß-oxidation [32]. However, growth on long chain and unsatured fatty acids in absence of catalase was surprising, since peroxisomes are required for their degradation [32], [33]. This latter result suggested either that the peroxide produced during the peroxisomal ß-oxidation was destroyed by enzymes other than typical catalases, or that, like in *N. crassa*, the first step of ß-oxidation was catalyzed by acyl-CoA dehydrogenases and consequently did not produce peroxide [34]–[37].

To test these hypotheses, we searched with BLASTP the *P. anserina* proteome for acyl-CoA oxidase and potential peroxisomal FA-CoA dehydrogenases. We found no gene encoding for acyl-CoA oxidase and 11 encoding putative FA-CoA dehydrogenases, including five with possible peroxisome function/localization (Pa_2_10530, Pa_2_12930, Pa_5_9600, Pa_5_4240 and Pa_2_12630). Absence of acyl-CoA oxidase and presence of several putative FA-CoA dehydrogenases strongly suggest that the first step of ß-oxidation is catalyzed by acyl-CoA dehydrogenase in *P. anserina* and hence does not produce H_2_O_2_.

### The Catalase-peroxidase 2 Activity is Necessary for Complex Biomass Assimilation as Lignocellulose but not for Cellulose Breakdown

*P. anserina* is usually grown on minimal medium containing dextrins as sole carbon source. However, it can also grow using cellulose or more complex biomass like lignocellulose [26]. We therefore tested the growth and reproduction ability of the catalase mutants on these carbohydrates as sole carbon sources.

**Figure 3:**
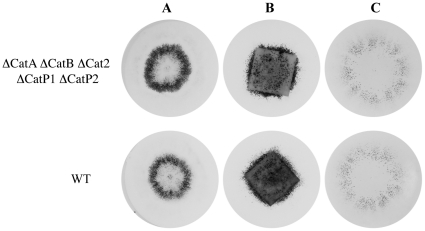
Phenotypes of wild type and the catalase quintuple mutant on medium with cellulose. Self-fertile mycelia of the strain devoid of catalase (ΔCatA ΔCatB ΔCat2 ΔCatP1 ΔCatP2) and of the wild-type strain (WT) were inoculated at the center of the plate on medium with (*A*) dextrins as sole carbon source, (*B*) on 3 cm×3 cm filter papers as sole carbon source, and (*C*) on medium with cellulose powder as sole carbon source. On the 3 media, after seven days of growth, WT, and the catalase mutants had differentiated numerous fructifications (the small black dots) mostly in a region surrounding the explant and no difference was observed between WT and the mutant.

First, we replaced dextrins by Whatman filter paper or cellulose powder. As it was previously shown that inability to efficiently scavenge cellulose had a drastic impact on the efficiency of perithecium production [38], we analyzed the number of perithecia produced by the catalase mutants on those two media. None of the mutants displayed sexual defects (Figure 3). Both number and development rate of fruiting bodies were indistinguishable from those of wild type. Furthermore, perithecia produced without delay a wild-type number of asci containing fully viable ascopores (n = 50).

**Figure 4:**
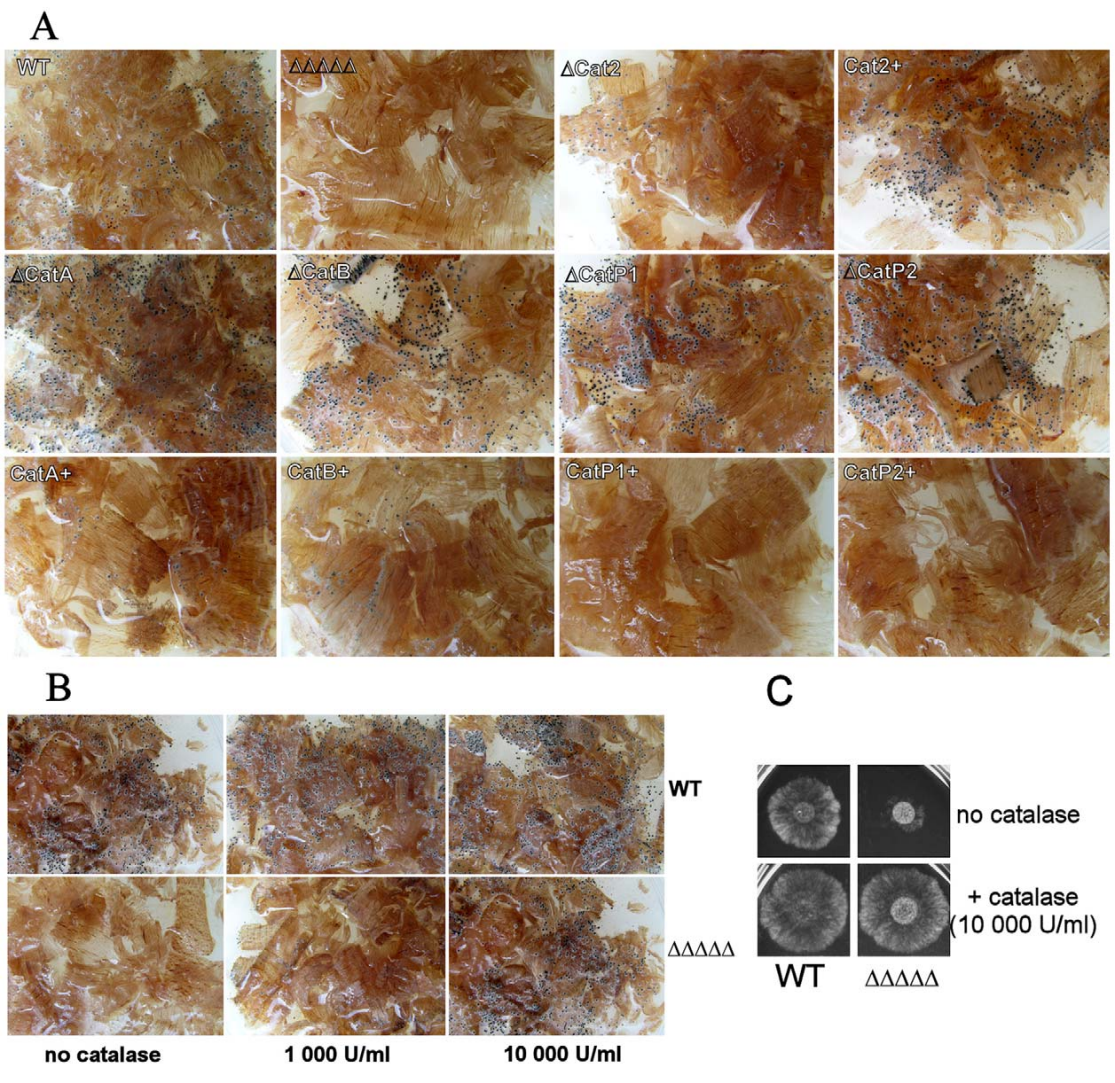
Phenotypes of wild type and catalase mutants on medium with wood shavings. (*A*) Mycelia of the single, quadruple and quintuple catalase mutants and wild type (WT) were inoculated at the center of the plate. After seven days of growth at which time the pictures were taken, WT differentiated perithecia (small black dots). The quintuple catalase (ΔΔΔΔΔ) mutant differentiates no fructification. The Δ*Cat2* single mutant strain differentiated fewer perithecia than WT and with a delay of 24 H, as evidenced by the smaller size of the perithecia. All the other single mutants (Δ*CatA*, Δ*CatB*, Δ*CatP1* and Δ*CatP2*) differentiated more fructification with 12 H of advance (seen by their larger size) in comparison to WT. The CAT2^+^ quadruple mutant (Δ*CatA* Δ*CatB* Δ*CatP1* Δ*CatP2*) showed a phenotype similar to WT. The other quadruple catalase mutants (CatA^+^: Δ*CatB* Δ*Cat2* Δ*CatP1*Δ*CatP2*; CatB^+^: Δ*CatA* Δ*Cat2* Δ*CatP1* Δ*CatP2*; CatP1^+^: Δ*CatA* Δ*CatB* Δ*Cat2* Δ*CatP2*; CatP2^+^: Δ*CatA* Δ*CatB* Δ*Cat2* Δ*CatP1*) differentiated less perithecia than the WT with delays of variable length. CatP1^+^ and CatP2^+^ perithecia are produced after nine day of growth and are not seen at seven day. (B) ΔΔΔΔΔ and WT were inoculated at the center of the plate in presence of 0, 1000 or 10 000 U/ml of bovine catalase. As in A, pictures were also taken at seven days of incubation. (C) ΔΔΔΔΔ and WT were inoculated on solid medium with lignin as sole carbon source without bovine catalase (Top) or with 10 000 U/ml of bovine catalase (bottom). The picture was taken after two days of growth.

Secondly, we tested the capacity of *P. anserina* catalase mutants to grow on wood shavings of *Guibourtia demeusi*. On this wood, wild-type strains grew as a spindly mycelium, but produced many perithecia. They were visible after seven days of growth and expelled viable ascospores two days after (Figure 4A). In contrast, the catalase mutants exhibited drastic defects when grown on the same shavings. The quintuple mutant displayed highly altered growth rates: among the 12 plates tested, six showed no growth at all and the six others exhibited extremely reduced growth. The quintuple mutant was also specifically defective in perithecia formation. (i) Only two of the 6 plates harboring mycelium showed perithecia. (ii) Their formation was delayed: 11 days of incubation was required instead of the seven days observed for wild type and (iii) perithecia contained only few ascospores (∼10% of the wild-type amount per perithecium). Thus, catalases play crucial roles for both growth and reproduction when wood is the sole carbon source.

To confirm the requirement of catalase activity to properly assimilate wood, wild-type and the quintuple mutant strains were inoculated on wood shaving medium supplemented with two concentrations of bovine catalase (1000 u/ml and 10000 u/ml). On this medium the wild-type strain grew and produced perithecia as on wood shavings alone (Figure 4B). Interestingly, the growth of the quintuple mutant was restored to a wild-type level and numerous perithecia containing viable ascospores were differentiated with the same kinetics as in the wild-type strain. Moreover, the number of perithecia produced by the mutant increased with the concentration of bovine catalase, but globally their number remained lower than the number of perithecia produced by the wild-type strain. Finally, no improvement was observed when heat inactivated catalase was used, showing that catalase activity was required for production of fruiting bodies on wood shavings.

To check whether catalases are needed for growth in the presence of lignin, the wild-type strain and the quintuple mutant were inoculated on medium with pure lignin as sole carbon source (Figure 4C). On this medium, wild type is able to grow [26], however, growth of the quintuple mutant was much altered (Figure 4C). Normal growth was restored by addition of bovine catalase, as seen for wood shavings (Figure 4C). All these data demonstrate that catalase activity is required for complex biomass utilization by *P. anserina*, such as wood shavings, probably by protecting cellular structures against the H_2_O_2_ generated by enzymes degrading lignin.

To specify which of the *P. anserina* catalases was required for complex biomass assimilation, the five single mutants (thus lacking a single catalase) and the five quadruple mutants (thus expressing only one of the catalases) were inoculated on wood shaving medium (6 plates for each strain). The results are presented in Figure 4A. All the mutants grew and produced ascospore-containing perithecia, but with different kinetics and efficiency. When compared to wild type, theΔ*Cat2* mutant displayed a delay of at least 24 hours for ascospore formation and produced fewer perithecia (∼60% less). Unexpectedly, the four other single mutants (Δ*CatA*, Δ*CatB*, Δ*CatP1* and Δ*CatP2*) not only produced more perithecia than wild type (∼50% more) but these perithecia were produced 12 hours earlier than wild-type perithecia (Figure 4A). The Δ*CatA* Δ*CatB* Δ*CatP1* Δ*CatP2* mutant strains expressing only catalase-peroxydase *Cat2* grew and produced fruiting bodies like wild type. In contrast, the four other quadruple mutants produced much less perithecia (∼80% less) and moreover showed delayed ascospore formation (Figure 4A). The defect was especially obvious in the mutants expressing CATP1 or CATP2 where perithecia were produced two days after wild type.

### ΔCatB and ΔCat2 Mutant Strains Exhibit an Increased Sensitivity to Peroxide Stress During Growth and Ascospore Germination

**Figure 5:**
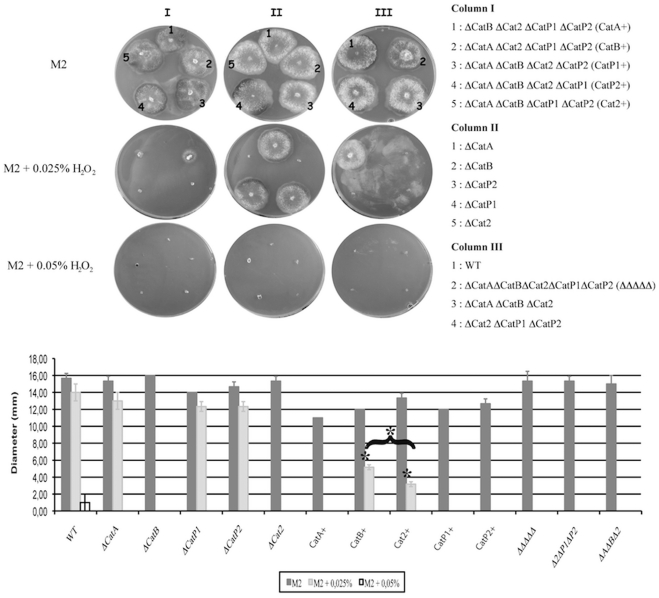
Sensitivity to hydrogen peroxide (H_2_O_2_) of wild type and the catalase mutant strains. Upper panel depicts the growth phenotypes. The strains were inoculated at 27°C on solid M2 medium without H_2_O_2_ (M2) or with 0.025% or 0.05% H_2_O_2_. Diameters (mm) of the thalli were measured after 48 hours. Lower panel: Corresponding histograms. The value for each strain is an average of 6 cultures and error bars represent standard deviation. Significant changes are indicated by an asterisk (p<0.001).

To assess the roles of the five catalases in the resistance to peroxide and other ROS, we tested the effect on mycelium growth of four inducers of oxidative stress: peroxide (H_2_O_2_), Tert-Butyl–hydroperoxide (an organic peroxide), uric acid for which the utilization as sole nitrogen source generates H_2_O_2_ [43] and Menadione that induces the production of superoxide (O_2_^.−^) inside the cell [39]–[43]. Only uric acid and H_2_O_2_ induced some growth defects in a subset of the single and multiple catalase mutants (Figure 5 and Table 1).

**Table 1:** Growth of the wild-type and catalase mutant strains on medium with different nitrogen source.

Strains genotype	Growth in cm after two days	
	Urea 8 mM	uric acid 8 mM
WT	1.6±0.2	1.7±0.3
Δ*CatA*	1.6±0.2	1.5±0.3
Δ*CatB*	1.7±0.2	0.9±0.3^a^
Δ*Cat2*	1.6±0.3	1.1±0.2^a^
Δ*CatP1*	1.6±0.3	1.6±0.1
Δ*CatP2*	1.5±0.2	1.5±0.1
Δ*CatA* Δ*CatB* Δ*Cat2* Δ*CatP1* Δ*CatP2*	1.6±0.2	0.2±0.2^a^

Each number is the mean value of 6 Petri dishes with the standard deviation.

a Significant value (p-value<0.01).

The lethal concentration of peroxide for wild type was 0.05%, as described [30]. This concentration was lethal for all catalase mutants (Figure 5). With 0.025% of H_2_O_2_ in the medium, the wild-type strain grew as on standard medium lacking peroxide. In contrast, the two Δ*CatB* and Δ*Cat2* single mutants were unable to grow. The three other single catalase mutants, Δ*CatA*, Δ*CatP1* and Δ*CatP2*, grew normally (Figure 5). Peroxide hypersensitivity was observed also for the quintuple mutant. This indicated that CATB and CAT2 were both needed to destroy the deleterious peroxide during vegetative growth and that the other catalases did not compensate for the lack of either CATB or CAT2.

Interestingly, the quadruple catalase mutant strains, expressing either only CATB or only CAT2 exhibited intermediate growth (Figure 5). While the single Δ*CatB* and Δ*Cat2* mutants did not grow in presence of 0.025% H_2_O_2,_ the Δ*CatA* Δ*Cat2* Δ*CatP1* Δ*CatP2* and Δ*CatA* Δ*CatB* Δ*CatP1* Δ*CatP2* quadruple mutants grew on this concentration (thalli diameter of respectively 5.2±0.3 and 3.2±0.3 cm after 48 hours), although the diameter of their thalli was significantly smaller than that in absence of H_2_O_2_ (respectively 12±0.1 and 13.3±0.6; Figure 5). These data suggested the existence of a mechanism of compensation for the lack of CAT2 or CATB in both mutants. However the trigger of his mechanism may be complex. For example, expressing only CATP1 and CATP2 (Δ*CatA* Δ*CatB* Δ*Cat2* mutants) or only CATA and CATB (Δ*Cat2* Δ*CatP1* Δ*CatP2* mutants) did not permit growth on 0.025% peroxide.

We then tested the growth of the wild-type strain, the quintuple catalase mutant and the five catalase mutants on medium uric acid as nitrogen source, instead of urea (Table 1). The wild-type strain grew as on all tested acid uric concentrations, like on standard medium with 8 mM urea. In contrast, the growth of the quintuple mutant strain was defective on medium with 8 mM acid uric (Table 1). To specify which of the *P. anserina* catalases was required for acid uric assimilation, the five single mutants (thus lacking a single catalase) were inoculated on 8 mM uric acid medium. Only the two Δ*CatB* and Δ*Cat2* single mutants display intermediate grow on this medium (Table 1). This indicated that CATB and CAT2 were both needed to destroy the deleterious peroxide produced by uric acid assimilation and that the other catalases could not compensate for the lack of either CATB or CAT2, as described above for resistance to peroxide.

We next analyzed the role of the catalases in defense against H_2_O_2_ during the sexual cycle, using the germination capacity of the ascospores produced by the five single catalase mutants and the quintuple catalase mutant as a test (Table 2). Ascospore germination is more sensitive to H_2_O_2_ than mycelium growth: when incubated at a concentration of 0.025% H_2_O_2_ neither wild-type nor mutant ascospores germinated (Table 2). After incubation with 0.005% H_2_O_2_, wild-type ascospores germinated with 100% efficiency, but only 29% of the ascospores of the quintuple Δ*CatA* Δ*CatB* Δ*Cat2* Δ*CatP1* Δ*CatP2* mutant germinated, showing a role of catalase in protecting ascospores. To decipher the role of each catalase, we analyzed the ascospore germination for each single mutant. Clearly, catalase-B (CATB) and to a lesser extent, the catalase-peroxidase 2 (CAT2) were the major actors of the defense system against H_2_O_2_ (Table 2). However, in 0.005% H_2_O_2,_ the germination rate of the two single Δ*Cat2* and Δ*CatB* mutants was intermediate between wild type and the quintuple mutant. This was further observed in the triple mutant ΔCatA ΔCatB ΔCat2 (65% germination) and the quintuple mutant (29%; n = 300 ascospores for each mutant). Therefore, although CAT2 and CATB have the major function in detoxifying H_2_O_2_ during ascospore germination, minor roles for the other catalases cannot be excluded.

**Table 2:** Germination of ascospores of catalase mutant strains in presence of peroxide.

Strains genotype	% of Germination on standard medium+H_2_O_2_		
	0%	0.005%	0.025%
WT	100 (120)	100 (300)	0 (150)
Δ*CatA*	97 (124)	97 (300)	0 (150)
Δ*CatB*	97 (132)	50 (300)	0 (150)
Δ*Cat2*	97 (124)	83 (300)	0 (150)
Δ*CatP1*	100 (110)	94 (300)	0 (150)
Δ*CatP2*	100 (120)	97 (300)	0 (150)
Δ*CatA* Δ*CatB* Δ*Cat2*	97 (120)	65 (300)	0 (150)
Δ*CatA* Δ*CatB* Δ*Cat2* Δ*CatP1* Δ*CatP2*	100 (120)	29 (300)	0 (150)

## Discussion

Our results show that the saprobic filamentous fungus *P. anserina* expresses five genes encoding four typical catalases and one peroxidase/catalase. More specifically, *CatB*, encoding a monofonctional large-subunit catalase and *Cat2*, encoding a peroxidase/catalase are constitutively expressed during vegetative growth with level much higher than the others. The CATB and CAT2 enzymes are the major factors involved in defense against peroxide in both mycelia and ascospores. In addition, while the catalases are not necessary for growth on glucose polymers (dextrins or cellulose), CAT2 plays a crucial role during vegetative growth and sexual development on complex biomass like wood shavings.

### CATB and CAT2 are Major Players in Cell Defense Against ROS

Among the five *P. anserina* catalase single mutants, only Δ*Cat2* and Δ*CatB* show a clear increase in sensibility to H_2_O_2_ during vegetative growth. Recently, Zintel and co-workers have obtained similar result for CATB. The authors reported that deletion of *CatB* increase the sensibility of the mutant strain against H_2_O_2_ whereas strains that over-express *CatB*, display an increase tolerance against H_2_O_2_ [29]. Similar results were obtained for *N. crassa cat-3* (orthologous to *CatB*) and *A. nidulans catA* and *catB* (orthologous to *P. anserina CatA* and *CatB*, respectively [39], [44]). This implies that these genes have conserved functions in H_2_O_2_ protection. Yet, the Δ*Cat2* and Δ*CatB* mutants exert hyphal interference, which is associated with an oxidative burst, and accumulate ROS like wild type without apparent damage (see *Supporting information*). This may be explained by the presence of redundant antioxidant systems. In *S. cerevisiae*, both the mitochondrial cytochrome c peroxidases and the glutathione redox system are antioxidant defenses that overlap with the one provided by the catalases [45], [46]. Other systems like thiol peroxidases, alternative oxidase or superoxide dismutase all present in *P. anserina* could also be implicated in redundancy by providing a complex but robust system to regulate hydrogen peroxide. The same redundant systems may be involved in the peculiar compensation that we have detected in the quadruple mutants lacking either CAT2 or CATB. Alternatively, increased expression of the remaining catalase may be involved.

### *P. anserina* Likely does not Produce H_2_O_2_ During Peroxisomal ß-oxidation

In *N. crassa* there is no catalase activity in the glyoxysomes, the specialized peroxisomes where fatty acid β-oxidation occurs, as the first stage of β-oxidation is catalyzed by an acyl-CoA dehydrogenase, which produces no H_2_O_2_ [34]–[37]. Our data suggest that the same pathway operates in *P. anserina*. The absence of H_2_O_2_ production by peroxisomal ß-oxidation does not support the hypothesis previously advanced to explain the growth defect on oleic acid of the *P. anserina pex2* mutants [47]. It was proposed that in such mutants, peroxisomal ß-oxidation is mis-targeted to the cytosol, where it produces hydrogen peroxide. We suggest that the growth defect of the *pex2* mutants is not linked to accumulation of cytoplasmic H_2_O_2_, but rather results from an accumulation of an intermediate product of the ß-oxidation or possibly to oleic acid *per se*, as put forward for similar *A. nidulans* mutants [48].

### *P. anserina* Catalases are not Required During the Life Cycle in Standard Laboratory Conditions

In absence of all five catalases the vegetative growth and the sexual reproduction of *P. anserina* is completely normal in standard condition. This is unexpected in view of numerous lines of evidence suggesting important roles of ROS during development [5]–[9], [49], [50]. The fact that *P. anserina* catalase mutants are not altered in their sexual reproduction, argues that either peroxide *per se* is not implicated, or that alternative pathways compensate for loss of catalase. An interesting third possibility is that developmental ROS, as those generated by NADPH oxidases, are not free in the cell but rather channeled through specific protein pathways and disulfide bond formation, as for example described for the Yap1 pathway of *S. cerevisiae*. Also, the developmental roles of catalases described specifically for conidia production in *N. crassa* may not be present in *P. anserina*, which does not differentiate asexual conidia [42], [44].

### Catalases are Required for Efficient Complex Biomass Assimilation in *P. anserina*

Lignocellulose is a major constituent of plant cell walls, which, in turn, is the most abundant source of carbon on land. Microbial, especially fungal, activity is responsible for most of the plant biomass hydrolysis, in part by oxidative processes in the presence of extracellular peroxidase and external H_2_O_2_ [51]. *P. anserina* can synthesize many secreted ROS-generating enzymes and is able to complete its life cycle with wood shavings or lignin as sole carbon [26], [17]. The data reported here establish that catalase activity is mandatory for complex biomass utilization and more precisely by allowing growth of *P. anserina* in the presence of lignin. The likeliest explanation is that catalases must degrade some of the peroxide produced for complex biomass breakdown, otherwise levels toxic to the fungus are reached. This is supported by the beneficial effect of adding bovine catalase on the growth and reproduction of the mutant lacking all its own enzymes. These results are consistent with the observation that one of the four catalase of the White Rot *Phanaerochaete chrysosporium* (protein 124398) is up-regulated during ligninolytic metabolism [52]. This catalase is associated with the outer envelope of the fungus and likely has a surveillance role against extracellular reactive oxygen species. Here, we provide genetic support for such model.

The *P. anserina* intracellular peroxidase/catalase CAT2 is the major catalase required for efficient growth on wood shavings. The fact that the CAT2 is also one of the two catalases implicated in mycelium defense against H_2_O_2_ suggests that CAT2 plays a role against the H_2_O_2_ that is produced in the medium and diffuses inside the cells. Interestingly, inactivation of the other catalase genes, in particular *CatB* gene which encodes the secreted catalase, results in an increased efficiency of wood shaving utilisation, as evidenced by faster and increased production of fruiting bodies. However, we cannot rule out that increased production is not due to actual increased assimilation, but by more efficient developmental ROS signaling specifically on wood shavings. Overall, these data nicely illustrate the antagonistic functions of H_2_O_2_ in complex biomass utilization. One the one hand, H_2_O_2_ participates to the degradation of complex biomass and thus allows more efficient use of this carbon source [16], [53]. But H_2_O_2_ is also a dangerous molecule responsible of cell damage and cell death. Thus, under ligninolytic condition, H_2_O_2_ must be tightly regulated to efficiently degrade plant biomass. Apparently, *P. anserina* does so by using five catalases of different families, which must be expressed in different compartments and must be regulated appropriately. Finally, these results suggest that the capacity of degradation of plant biomass by fungi could be increased by modulation of catalase activity. This finding is an important step towards understanding how fungi are able to use wood as growth substrate, the role of fungi in plant biomass degradation and provides therefore new insights into potential engineering applications.

## Materials and Methods

### Strains and Methods

Life cycle, media and genetic methods for *P. anserina* are described in http://podospora.igmors.u-psud.fr. Transformation was carried out as described previously [54]. Transformants were selected on medium containing 20 µg/ml phleomycin, 100 µg/ml hygromycin B or 75 µg/ml nourseothricin. The M2 standard minimal synthetic medium contains dextrins (0.5%) and Urea (8 mM). Dextrins were replaced with various fatty acids, cellulosic materials (3 cm×3 cm Whatman filter paper or cellulose powder), lignin (Sigma cat # 471003) and *Guibourtia demeusi* wood shavings as carbon sources. Urea was replace with Uric acid as nitrogen source. Bovine catalase was purchased from Sigma-Aldrich Co, St Louis MO (ref. 9001-05-2) and dissolved in water. It was inactivated by boiling 5 minutes at 100°C. Oxidative stress experiments were performed on M2 containing Menadione (10^−4^, 10^−5^, 10^−6^ M), t-butyl-hydroxyperoxide (10^−5^, 10^−6^, 10^−7^ M), and H_2_O_2_ (0,1%, 0,05%, 0,025%, 0,001%). Longevity was measured as in [55]. CG (Crippled Growth) was evidenced as in [56], hyphal interference as in [30]. DAB and NBT assays were performed as in [7] and estimation of microconidial production and fertilization ability as in [57].

### Deletion of Catalase Genes

Null mutants were generated by gene substitution using methods previously described for the *echA* gene [32]. For *CatP2* and *CatP1* genes, the 5′ and 3′ flanking sequences were amplified by PCR and introduced in a plasmid containing the *hph* gene (conferring resistance to hygromycin B), for the *Cat2* and *CatB* genes in a plasmid conferring resistance to nourseothricin and for the *CatA* gene in a plasmid conferring resistance to phleomycin. Transformants carrying a deleted allele were confirmed by PCR and Southern blotting. All possible combinations of double, triple and quadruple mutants as well as the quintuple mutant completely devoid of its five catalases were constructed by genetic crosses. The genotypes of all multiple mutants were confirmed by PCR (data not shown).

### DNA, RNA and qPCR Procedures

Total *P. anserina* DNA required for both PCR and Southern blot experiments was extracted using a miniprep method [58]. Total RNA was isolated from mycelia as described in Brun *et al.* [9]. The expressions of catalase genes were monitored by Real-time quantitative PCR as previously reported [32]. The primer sequences used for Real Time PCR are given upon request.

## Supporting Information

Figure S1 **Phylogenetic analyses of the** ***P. anserina*** **catalases.** (A) the peroxidase/catalase and (B) the four catalases relationships are shown by a ML-tree. The molecular evolutionary relationships was analyzed within a group of 17 Ascomycota, 2 Basidiomycota (*C. neoformans* and *U. maydis*) and one Mucoromycotina (*R. oryzae*) by application of three distinct phylogenetic methods/(i) NJ (neighbour-joining) distance method, (ii) MP (maximum parsimony) method, and (iii) ML (maximum likelihood) method. The three approaches gave very similar tree topologies. The ML-tree is displayed with statistical support from 100 bootstrap replications. For clarity in visualizing the tree, only statistical values above 50 are presented.
(TIF)

Figure S2 **Crippled Growth Assay of the quintuple mutant devoid of catalase as compared to wild type.** Left is depicted the experimental setup. A culture is grown for 7 days on M2 medium. Four explants are taken at various distances from the growing edge (indicated as white squares from 1 to 4) from the growing edge (black square) and replicated onto M2 medium (reference medium as in top circle) or M2 medium supplemented with 5 g/l of yeast extract (as in bottom circle). Actual plates are on the right. The same results are obtained for wild type (middle) and mutants devoid of all their catalases (right). On M2 (top circles) no crippled growth is observed. On M2 supplemented with yeast extract (WT bottom circles), Crippled Growth develops as a flat and spindly mycelium with pigment accumulation in culture originating from stationary phase explants (CG). In contrast, explants taken from the growing edge present a normal growth (NG).
(TIF)

Figure S3 **Comparison of wild type and catalase mutant phenotypes for constitutive ROS secretion and hyphal interference.** (A) Peroxide and superoxide accumulation patterns in wild type (WT) and the quintuple catalase mutants. Superoxide is detected by treatment with nitroblue tetrazolium to yield a blue precipitate (left column). Peroxide accumulation is detected by treatment with diaminobenzidine and peroxidase as a reddish precipitate (right column). Both staining assays were made on 72 hours old cultures during 2 hours (peroxide or superoxide). The graphs below the plates show quantification along a diameter (x-axis). The y-axis is the intensity in an arbitrary unit. The arrows indicate the zones of highest secretion, along a ring for superoxide (left) and at the center for peroxide (right). (B) Hyphal Interference of different catalase mutant strains against *P. chrysogenum*. Top row shows the oxidative burst (as seen by intense DAB precipitation, arrow) in wild type and the indicated catalase mutants after they contact *P. chrysogenum* for one day. Bottom row illustrates the accumulation of dead *P. chrysogenum* hyphae (vizualised by intense Trypan Blue staining, arrow) when confronted with WT and catalase mutants. ΔΔΔΔΔ = Δ*CatA* Δ*CatB* Δ*Cat2* Δ*CatP1* Δ*CatP2*; A^+^ = Δ*CatB* Δ*Cat2* Δ*CatP1* Δ*CatP2*; B^+^ = Δ*CatA* Δ*Cat2* Δ*CatP1* Δ*CatP2*; 2^+^ = Δ*CatA* Δ*CatB* Δ*CatP1* Δ*CatP2*; P1^+^ = Δ*CatA* Δ*CatB* Δ*Cat2* Δ*CatP2*; P2^+^ = Δ*CatA* Δ*CatB* Δ*Cat2* Δ*CatP1*.
(TIF)

Table S1 Estimation of microconidial production of the quintuple mutant strain.
(DOC)

Table S2 Growth rate and Life span of the wild-type and mutant strains at 27°C.
(DOC)

Table S3 Growth rate of the wild-type and quintuple mutant at 11°C and 37°C.
(DOC)

Text S1 Supplementary results.
(DOC)
