# Supplementary figures and images for: Wood Utilization Is Dependent on Catalase Activities in the Filamentous Fungus Podospora anserina
Source: PLoS One. 2012 Apr 27;7(4):e29820. doi: 10.1371/journal.pone.0029820 (PMC3338752; doi:10.1371/journal.pone.0029820)

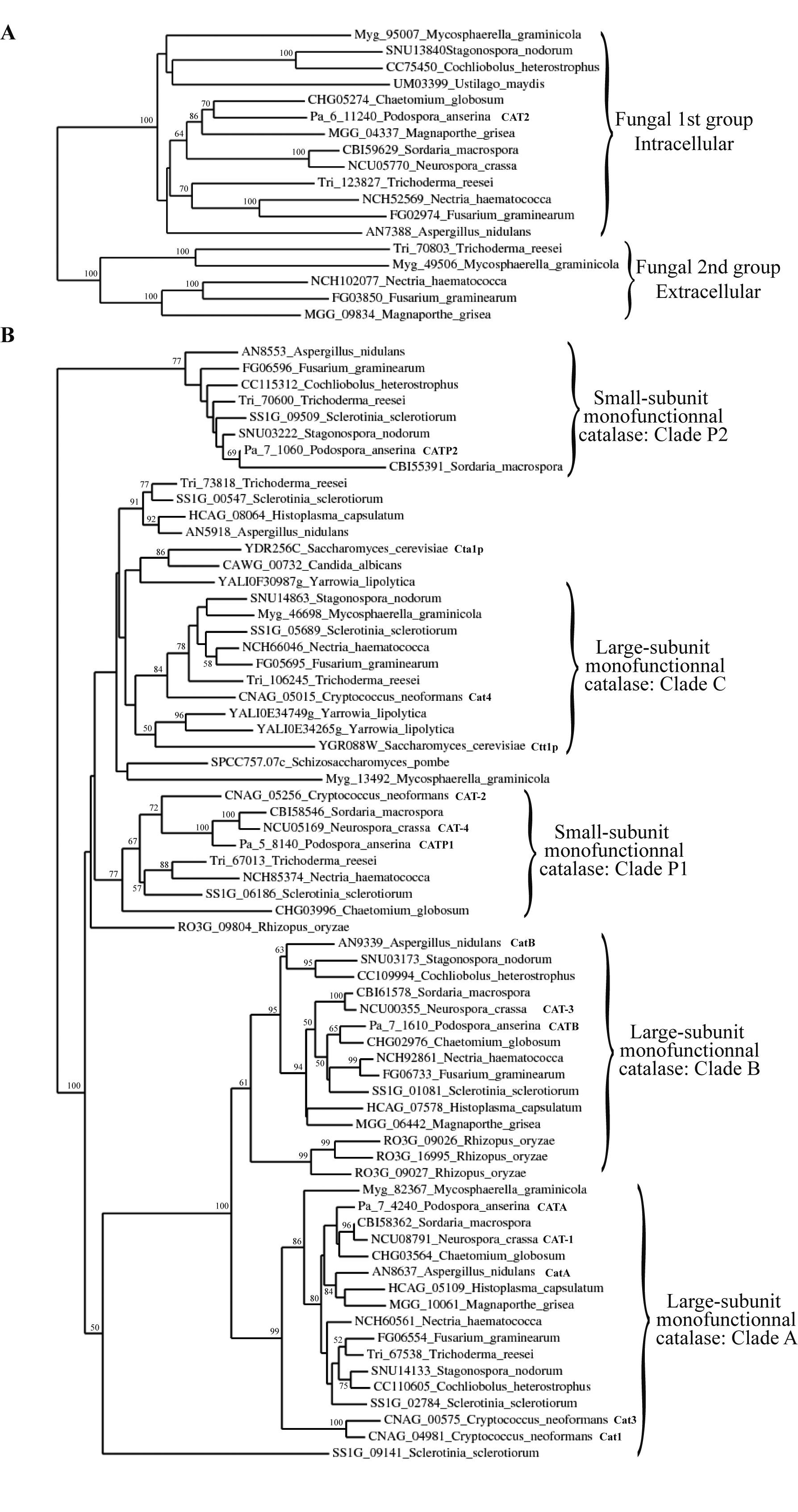

Supplement: Figure S1 — Phylogenetic analyses of the P. anserina catalases. (A) the peroxidase/catalase and (B) the four catalases relationships are shown by a ML-tree. The molecular evolutionary relationships was analyzed within a group of 17 Ascomycota, 2 Basidiomycota (C. neoformans and U. maydis) and one Mucoromycotina (R. oryzae) by application of three distinct phylogenetic methods/(i) NJ (neighbour-joining) distance method, (ii) MP (maximum parsimony) method, and (iii) ML (maximum likelihood) method. The three approaches gave very similar tree topologies. The ML-tree is displayed with statistical support from 100 bootstrap replications. For clarity in visualizing the tree, only statistical values above 50 are presented. (TIF) [file pone.0029820.s001.tif]

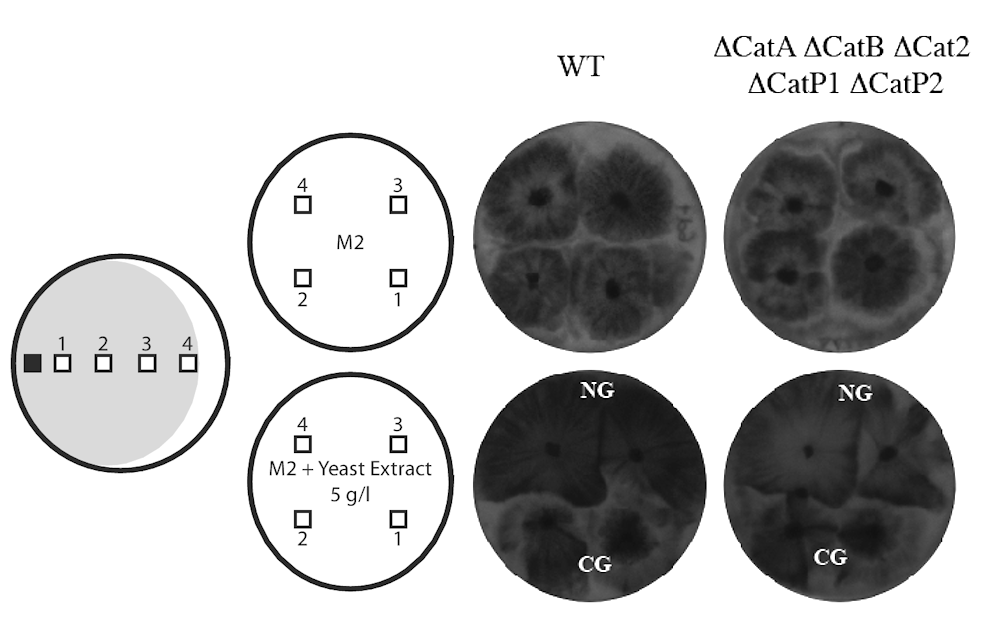

Supplement: Figure S2 — Crippled Growth Assay of the quintuple mutant devoid of catalase as compared to wild type. Left is depicted the experimental setup. A culture is grown for 7 days on M2 medium. Four explants are taken at various distances from the growing edge (indicated as white squares from 1 to 4) from the growing edge (black square) and replicated onto M2 medium (reference medium as in top circle) or M2 medium supplemented with 5 g/l of yeast extract (as in bottom circle). Actual plates are on the right. The same results are obtained for wild type (middle) and mutants devoid of all their catalases (right). On M2 (top circles) no crippled growth is observed. On M2 supplemented with yeast extract (WT bottom circles), Crippled Growth develops as a flat and spindly mycelium with pigment accumulation in culture originating from stationary phase explants (CG). In contrast, explants taken from the growing edge present a normal growth (NG). (TIF) [file pone.0029820.s002.tif]

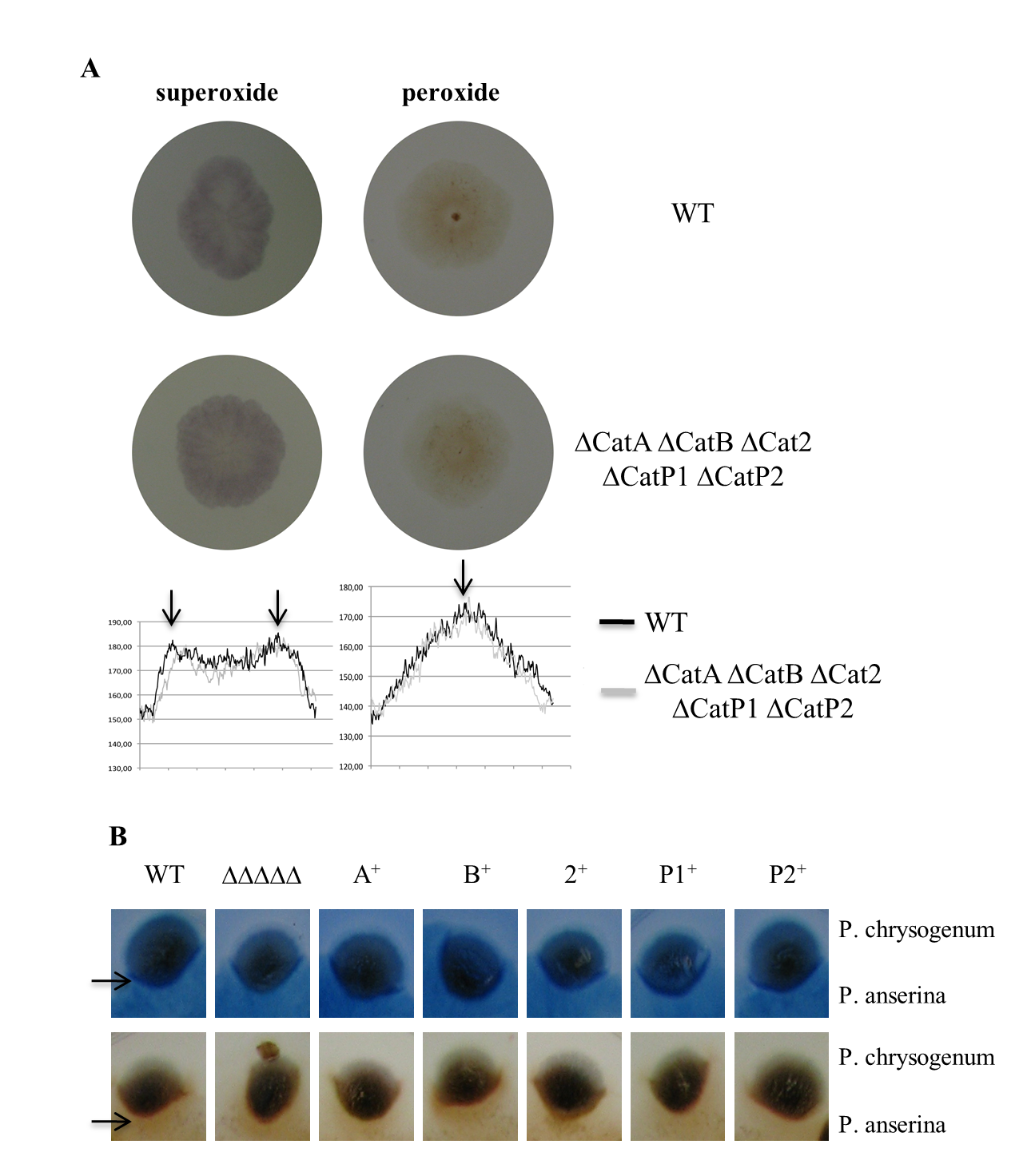

Supplement: Figure S3 — Comparison of wild type and catalase mutant phenotypes for constitutive ROS secretion and hyphal interference. (A) Peroxide and superoxide accumulation patterns in wild type (WT) and the quintuple catalase mutants. Superoxide is detected by treatment with nitroblue tetrazolium to yield a blue precipitate (left column). Peroxide accumulation is detected by treatment with diaminobenzidine and peroxidase as a reddish precipitate (right column). Both staining assays were made on 72 hours old cultures during 2 hours (peroxide or superoxide). The graphs below the plates show quantification along a diameter (x-axis). The y-axis is the intensity in an arbitrary unit. The arrows indicate the zones of highest secretion, along a ring for superoxide (left) and at the center for peroxide (right). (B) Hyphal Interference of different catalase mutant strains against P. chrysogenum. Top row shows the oxidative burst (as seen by intense DAB precipitation, arrow) in wild type and the indicated catalase mutants after they contact P. chrysogenum for one day. Bottom row illustrates the accumulation of dead P. chrysogenum hyphae (vizualised by intense Trypan Blue staining, arrow) when confronted with WT and catalase mutants. ΔΔΔΔΔ = ΔCatA ΔCatB ΔCat2 ΔCatP1 ΔCatP2; A+ = ΔCatB ΔCat2 ΔCatP1 ΔCatP2; B+ = ΔCatA ΔCat2 ΔCatP1 ΔCatP2; 2+ = ΔCatA ΔCatB ΔCatP1 ΔCatP2; P1+ = ΔCatA ΔCatB ΔCat2 ΔCatP2; P2+ = ΔCatA ΔCatB ΔCat2 ΔCatP1. (TIF) [file pone.0029820.s003.tif]
