# Supplementary material for: Wood Utilization Is Dependent on Catalase Activities in the Filamentous Fungus Podospora anserina
Source: PLoS One. 2012 Apr 27;7(4):e29820. doi: 10.1371/journal.pone.0029820 (PMC3338752; doi:10.1371/journal.pone.0029820)
Supplement: Table S1 — Estimation of microconidial production of the quintuple mutant strain. (DOC) [file pone.0029820.s004.doc]

**Table S1**

Estimation of microconidial production of the quintuple mutant strain

| **Strain genotypes** | **No of microconidia ^a^** | **No of perithecia produced by fertilization of wild-type strain** |
| --- | --- | --- |
| WT | 2.8 10^6^ ±0.3 | 294 ±10 |
| *∆CatA ∆CatB ∆Cat2 ∆CatP1 ∆CatP2* | 3 10^6^ ±0.2 | 280 ±30 |

**a.** Each number is the mean value of three Petri dishes with the standard deviation

**b.** Perithecia were counted after fertilization of the wild-type strain used as the female partner with 1 ml of microconidia suspension after dilution (300 microconidia /ml) from the relevant strain. The numbers are the mean values of three Petri dishes.
