# Supplementary material for: Wood Utilization Is Dependent on Catalase Activities in the Filamentous Fungus Podospora anserina
Source: PLoS One. 2012 Apr 27;7(4):e29820. doi: 10.1371/journal.pone.0029820 (PMC3338752; doi:10.1371/journal.pone.0029820)
Supplement: Table S2 — Growth rate and Life span of the wild-type and mutant strains at 27°C. (DOC) [file pone.0029820.s005.doc]

**Table S2**

Growth rate and Life span of the wild-type and mutant strains at 27 °C.

| **Strain genotypes** | **Growth rate (cm day^-1^) ± standard deviation** | | **Life span (cm) ± standard deviation** | |
| --- | --- | --- | --- | --- |
|  | *mat*+ | *mat*- | *mat*+ | *mat*- |
| WT | 0.69 ±0.06 | 0.69 ±0.07 | 10.8 ±1 | 10.5 ±1.1 |
| *∆CatA* | 0.71 ±0.05 | 0.7 ±0.04 | 10.3 ±0.9 | 10.4 ±1.3 |
| *∆CatB* | 0.69 ±0.06 | 0.7 ±0.07 | 9.4 ±1.1 | 9.1 ±0.5 |
| *∆Cat2* | 0.69 ±0.06 | 0.69 ±0.07 | 8.6 ±1.2 | 8.6 ±2.2 |
| *∆CatP1* | 0.69 ± 0.05 | 0.69 ±0.08 | 8.2 ±2.4 | 8.2 ±2.1 |
| *∆CatP2* | 0.7 ±0.06 | 0.72 ±0.02 | 8.5 ± 3.1 | 8.7 ±0.5 |
| *∆CatA ∆CatB ∆Cat2 ∆CatP1 ∆CatP2* | 0.71 ±0.07 | 0.74 ±0.06 | 8 ±0.8 | 8.5 ±0.6 |
