# Supplementary material for: Wood Utilization Is Dependent on Catalase Activities in the Filamentous Fungus Podospora anserina
Source: PLoS One. 2012 Apr 27;7(4):e29820. doi: 10.1371/journal.pone.0029820 (PMC3338752; doi:10.1371/journal.pone.0029820)
Supplement: Table S3 — Growth rate of the wild-type and quintuple mutant at 11°C and 37°C. (DOC) [file pone.0029820.s006.doc]

**Table S3**

Growth rate of the wild-type and quintuple mutant at 11°C and 37 °C

| **Strains genotype** | **11 °C** | | | | **37 °C** | | | |
| --- | --- | --- | --- | --- | --- | --- | --- | --- |
|  | *mat*+ | | *mat*- | | *mat*+ | | *mat*- | |
| WT | | 0.16 ±0.01 | | 0.18 ±0.03 | | 0.5 ±0.01 | | 0.7 ±0.02 |
| *∆CatA ∆CatB ∆Cat2 ∆CatP1 ∆CatP2* | | 0.16 ±0.02 | | 0.18 ±0.05 | | 0,5 ±0.03 | | 0.75 ±0.005 |
