# Supplementary material for: Wood Utilization Is Dependent on Catalase Activities in the Filamentous Fungus Podospora anserina
Source: PLoS One. 2012 Apr 27;7(4):e29820. doi: 10.1371/journal.pone.0029820 (PMC3338752; doi:10.1371/journal.pone.0029820)
Supplement: Text S1 — Supplementary results. (DOC) [file pone.0029820.s007.doc]

Anne Bourdais^1,2,3^, Frederique Bidard^1,2^, Denise Zickler^1,2^, Veronique Berteaux-Lecellier^1,2,4^, Philippe Silar^1,2,5^ and Eric Espagne^1,*^

^1^ Univ Paris-Sud, Institut de Génétique et Microbiologie, UMR 8621, ORSAY, F-91405.
^2^ CNRS, ORSAY, F-91405.

^3^ CNRS, UMR 6061, Institut Génétique et Développement de Rennes, F-35043 Rennes, France ; Université Rennes 1, UEB, IFR 140, Faculté de Médecine, F-35043 Rennes, France.

^4^ Laboratoire d'Excellence « CORAIL », USR 3278 CNRS-EPHE, CRIOBE, BP 1013, 98729 Moorea, French Polynesia.

^5^ Univ Paris Diderot, Sorbonne Paris Cité, UFR des Sciences du Vivant, 75205 Paris CEDEX 13 France.

^*^To whom correspondence may be addressed:

Eric Espagne, PhD

Univ. Paris-Sud 11, CNRS, UMR8621,

Institut de Génétique et Microbiologie,

91405 Orsay, France.

E-mail: [eric.espagne@igmors.u-psud.fr](mailto:eric.espagne@igmors.u-psud.fr)

Phone: 33 (1) 69.15.46.47 ; Fax : 33 (1) 69.15.70.06

**Catalases are dispensable during the Vegetative Cycle:** We investigated the function of the different catalases during vegetative growth under standard conditions. Growth rate and life span were precisely quantified for the five single mutants and for the quintuple mutant (Table S1). All investigated strains showed a wild-type growth rate (0.7 cm /day). Moreover, senescence was normally expressed by an increase of mycelial pigmentation and death of apical cells (reviewed in Jamet-Vierny [1]). Senescence appeared after 9 + 1 cm of growth in race tubes in the mutants as compared to 10 + 1 cm for wild type.

To test if the catalase mutants are sensitive to an inappropriate activation of the MAPK pathways, we analyzed the development of an epigenetic cell degeneration phenomenon, called crippled growth (CG), which appears as consequence of an inappropriate activation of a MAPK pathway [2]. This CG phenotype is characterized by sectors of poor growth displaying an over-pigmented and female sterile mycelium. Involvement of the NADPH oxidase *PaNox1* in the development of CG suggests that cellular level of ROS could be important in the propagation of the CG phenotype [3]. In wild-type strains, CG is never observed on standard medium but is observed on medium supplemented with yeast extract [4]. All the catalase mutant strains behave like wild type (Figure S2). Those results indicate that catalases are dispensable during the vegetative phase in various growth conditions.

**Catalases are dispensable during the Sexual Cycle.** We checked the role of catalases during all steps of sexual reproduction, starting with fertilization. To do so, we analyzed the number of conidia formed on the quintuple mutant mycelium and tested their capacity to fertilized the wild-type strain by counting the number of formed perithecia (fruiting bodies). Data reported in Table S1 show that both conidial production (evaluated by the number of conidia per plate) and fertilization capacity (number of perithecia produced after fertilization with 300 microconidia) are normal. Moreover, the number of perithecia (5000-6000 per Petri dish, n = 6) formed in a homozygous mutant cross (fertilization of the quintuple mutant by it self) was similar to number formed in wild-type crosses. Furthermore, perithecial development was completely normal: morphology, number of asci (about 100), meiosis and ascospore production were identical to wild type. All the other investigated mutants behaved as wild type and the quintuple mutants.

**Catalase mutants do not exhibit modification of ROS secretion or defense capacity against competitor fungi.** *P. anserina* mycelium is known to present constitutive accumulation of ROS or redox activity [5]. Therefore, we evaluated superoxide and peroxide accumulation of the mycelia after 48 h of growth in the quintuple mutant strains as compared to wild type (Figure S3A). ROS/Redox activities can be measured by diaminobenzidine (DAB) and nitroblue tetrazolium (NBT) assays (3). The two strains showed the same pattern of precipitate accumulation for both tests. These results indicate that absence of four or all five catalases did not modify the level and the pattern of ROS/redox activity during vegetative growth.

*P.* *anserina* grows on dung in nature, and therefore is always surrounded by other fungi. It exerts hyphal interference against them [6]. This defense reaction, which exists only in non-self confrontation, can be evidenced either by the revelation of the oxidative burst at the contact zone (DAB staining) or by the coloration of dead cells at the confrontation area by Trypan Blue staining (6). In order to test if catalase activities are implicated in this defense system, we confronted *P.* *anserina* to *Penicillium chrysogenum*. In this assay, there was no difference in peroxide accumulation (DAB staining) or in cell death (Trypan Blue staining) in the catalase mutants when compared to wild type (Figure S3B). Therefore catalases are dispensable for *P.* *anserina* defense by hyphal interference.

Reference

1. Jamet-Vierny C, Rossignol M, Haedens V, Silar P (1999) What triggers senescence in Podospora anserina? Fungal Genet Biol 27: 26–35. doi:10.1006/fgbi.1999.1127.

2. Silar P, Haedens V, Rossignol M, Lalucque H (1999) Propagation of a novel cytoplasmic, infectious and deleterious determinant is controlled by translational accuracy in Podospora anserina. Genetics 151: 87–95.

3. Malagnac F, Lalucque H, Lepère G, Silar P (2004) Two NADPH oxidase isoforms are required for sexual reproduction and ascospore germination in the filamentous fungus Podospora anserina. Fungal Genet Biol 41: 982–997. doi:10.1016/j.fgb.2004.07.008.

4. Haedens V, Malagnac F, Silar P (2005) Genetic control of an epigenetic cell degeneration syndrome in Podospora anserina. Fungal Genet Biol 42: 564–577. doi:10.1016/j.fgb.2005.03.011.

5. Malagnac F, Bidard F, Lalucque H, Brun S, Lambou K, et al. (2008) Convergent evolution of morphogenetic processes in fungi: Role of tetraspanins and NADPH oxidases 2 in plant pathogens and saprobes. Commun Integr Biol 1: 180–181.

6. Silar P (2005) Peroxide accumulation and cell death in filamentous fungi induced by contact with a contestant. Mycol Res 109: 137–149.

**Supporting Figure legends**

**Figure S1. Phylogenetic analyses of the *P. anserina* catalases.**

(A) the peroxidase/catalase and (B) the four catalases relationships are shown by a ML-tree. The molecular evolutionary relationships was analyzed within a group of 17 Ascomycota, 2 Basidiomycota (*C. neoformans* and *U. maydis*) and one Mucoromycotina (*R. oryzae*) by application of three distinct phylogenetic methods/ (i) NJ (neighbour-joining) distance method, (ii) MP (maximum parsimony) method, and (iii) ML (maximum likelihood) method. The three approaches gave very similar tree topologies. The ML-tree is displayed with statistical support from 100 bootstrap replications. For clarity in visualizing the tree, only statistical values above 50 are presented.

**Figure S2. Crippled Growth Assay of the quintuple mutant devoid of catalase as compared to wild type.**

Left is depicted the experimental setup. A culture is grown for 7 days on M2 medium. Four explants are taken at various distances from the growing edge (indicated as white squares from 1 to 4) from the growing edge (black square) and replicated onto M2 medium (reference medium as in top circle) or M2 medium supplemented with 5g/l of yeast extract (as in bottom circle). Actual plates are on the right. The same results are obtained for wild type (middle) and mutants devoid of all their catalases (right). On M2 (top circles) no crippled growth is observed. On M2 supplemented with yeast extract (WT bottom circles), Crippled Growth develops as a flat and spindly mycelium with pigment accumulation in culture originating from stationary phase explants (CG). In contrast, explants taken from the growing edge present a normal growth (NG).

**Figure S3. Comparison of wild type and catalase mutant phenotypes for constitutive ROS secretion and hyphal interference.**

(A) Peroxide and superoxide accumulation patterns in wild type (WT) and the quintuple catalase mutants. Superoxide is detected by treatment with nitroblue tetrazolium to yield a blue precipitate (left column). Peroxide accumulation is detected by treatment with diaminobenzidine and peroxidase as a reddish precipitate (right column). Both staining assays were made on 72 hours old cultures during 2 hours (peroxide or superoxide). The graphs below the plates show quantification along a diameter (x-axis). The y-axis is the intensity in an arbitrary unit. The arrows indicate the zones of highest secretion, along a ring for superoxide (left) and at the center for peroxide (right). (B) Hyphal Interference of different catalase mutant strains against *P. chrysogenum*. Top row shows the oxidative burst (as seen by intense DAB precipitation, arrow) in wild type and the indicated catalase mutants after they contact *P. chrysogenum*  for one day. Bottom row illustrates the accumulation of dead *P. chrysogenum* hyphae (vizualised by intense Trypan Blue staining, arrow) when confronted with WT and catalase mutants. *ΔΔΔΔΔ =  ΔCatA ΔCatB ΔCat2 ΔCatP1 ΔCatP2*; A^+^= *ΔCatB ΔCat2 ΔCatP1 ΔCatP2*; B^+^ = *ΔCatA ΔCat2 ΔCatP1 ΔCatP2*; 2^+^= *ΔCatA ΔCatB ΔCatP1 ΔCatP2*; P1^+^=* ΔCatA ΔCatB ΔCat2 ΔCatP2*; P2^+^= *ΔCatA ΔCatB ΔCat2 ΔCatP1*.

**TABLE S1**

**Estimation of microconidial production of the quintuple mutant strain**

| **Strain genotypes** | **No of microconidia ^a^** | **No of perithecia produced by fertilization of wild-type strain** |
| --- | --- | --- |
| WT | 2.8 10^6^ ±0.3 | 294 ±10 |
| *ΔCatA ΔCatB ΔCat2 ΔCatP1 ΔCatP2* | 3 10^6^ ±0.2 | 280 ±30 |

**a.** Each number is the mean value of three Petri dishes with the standard deviation

**b.** Perithecia were counted after fertilization of the wild-type strain used as the female partner with 1 ml of microconidia suspension after dilution (300 microconidia /ml) from the relevant strain. The numbers are the mean values of three Petri dishes.

**TABLE S2**

Growth rate and Life span of the wild-type and mutant strains at 27 °C.

| **Strain genotypes** | **Growth rate (cm day^-1^) ± standard deviation** | | **Life span (cm) ± standard deviation** | |
| --- | --- | --- | --- | --- |
|  | *mat*+ | *mat*- | *mat*+ | *mat*- |
| WT | 0.69 ±0.06 | 0.69 ±0.07 | 10.8 ±1 | 10.5 ±1.1 |
| *ΔCatA* | 0.71 ±0.05 | 0.7 ±0.04 | 10.3 ±0.9 | 10.4 ±1.3 |
| *ΔCatB* | 0.69 ±0.06 | 0.7 ±0.07 | 9.4 ±1.1 | 9.1 ±0.5 |
| *ΔCat2* | 0.69 ±0.06 | 0.69 ±0.07 | 8.6 ±1.2 | 8.6 ±2.2 |
| *ΔCatP1* | 0.69 ± 0.05 | 0.69 ±0.08 | 8.2 ±2.4 | 8.2 ±2.1 |
| *ΔCatP2* | 0.7 ±0.06 | 0.72 ±0.02 | 8.5 ± 3.1 | 8.7 ±0.5 |
| *ΔCatA ΔCatB ΔCat2 ΔCatP1 ΔCatP2* | 0.71 ±0.07 | 0.74 ±0.06 | 8 ±0.8 | 8.5 ±0.6 |

**TABLE S3**

Growth rate of the wild-type and quintuple mutant at 11°C and 37 °C

| **Strains genotype** | **11 °C** | | | | **37 °C** | | | |
| --- | --- | --- | --- | --- | --- | --- | --- | --- |
|  | *mat*+ | | *mat*- | | *mat*+ | | *mat*- | |
| WT | | 0.16 ±0.01 | | 0.18 ±0.03 | | 0.5 ±0.01 | | 0.7 ±0.02 |
| *ΔCatA ΔCatB ΔCat2 ΔCatP1 ΔCatP2* | | 0.16 ±0.02 | | 0.18 ±0.05 | | 0,5 ±0.03 | | 0.75 ±0.005 |
